# Movement efficiency in survivors of childhood acute lymphoblastic leukemia: a report from the St. Jude lifetime cohort study

**DOI:** 10.1007/s11764-024-01550-1

**Published:** 2024-02-03

**Authors:** Aron Onerup, Sedigheh Mirzaei S., Matthew D. Wogksch, Chelsea G. Goodenough, Genevieve Lambert, Yadav Sapkota, Daniel A. Mulrooney, Melissa M. Hudson, Lisa M. Jacola, Kirsten K. Ness

**Affiliations:** 1 Department of Epidemiology and Cancer Control, St. Jude Children´s Research Hospital, Memphis, TN USA; 2 Department of Pediatrics, Institute of Clinical Sciences, Sahlgrenska Academy, University of Gothenburg, Gothenburg, Sweden; 3 Department of Biostatistics, St Jude Children´s Research Hospital, Memphis, TN USA; 4 Department of Oncology, St Jude Children´s Research Hospital, Memphis, TN USA; 5 Department of Psychology, St Jude Children´s Research Hospital, Memphis, TN USA

**Keywords:** Lifestyle, Acute lymphoblastic leukemia, Childhood cancer, Survivorship, Epidemiology

## Abstract

**Purpose:**

Movement efficiency, a measure of neuromuscular biomechanics, may be modified by physical activity. We aimed to assess the risk of and risk factors for low movement efficiency in survivors of childhood acute lymphoblastic leukemia (ALL).

**Methods:**

Participants underwent an assessment of activity energy expenditure (AEE) with actigraphy, and the gold standard doubly labeled water, where the differences between elimination rates of oxygen and hydrogen from body water are evaluated over a week. Movement efficiency was assessed using the raw residuals of a linear regression between AEEs from accelerometers and doubly labeled water. Elastic-net logistic regressions were used to identify demographic, treatment, and functional variables associated with movement efficiency.

**Results:**

The study cohort included 256 non-cancer controls and 302 ALL survivors (48% female), categorized as efficient (*N* = 24), normal (*N* = 245), or inefficient (*N* = 33) based on their movement efficiency. There was no difference in the odds for poor movement efficiency between survivors (*n* = 33, 10.9%) compared to controls (*n* = 23, 9.0%, odds ratio [OR]: 1.19, 95% confidence interval [CI]: 0.67, 2.10; *p* = 0.55). In survivors, neuropathy was associated with a higher risk of being inefficient compared to efficient (OR 4.30, 95% CI 1.03–17.96), while obesity (≥ 30 kg/m^2^) had a protective association (OR 0.18, 95% CI 0.04–0.87).

**Conclusions:**

Neuropathy was associated with a higher risk of poor movement efficiency in survivors of childhood ALL.

**Implications for cancer survivors:**

These results further highlight impairments associated with treatment-induced neuropathy in survivors of childhood ALL.

**Supplementary Information:**

The online version contains supplementary material available at 10.1007/s11764-024-01550-1.

## Background

Increased survival rates for several types of childhood cancer during the last decades have resulted in a rapidly increasing population of childhood cancer survivors, with five-year overall survival after a diagnosis of childhood acute lymphoblastic leukemia (ALL) exceeding 94% with current protocols [1]. However, survivors of childhood ALL have increased risk for chronic health conditions, neurocognitive deficits, and premature death [2–4]. ALL survivors also have higher proportions of exercise intolerance and unfavorable body composition when compared to peers of similar age [5].

**Fig. 1:**
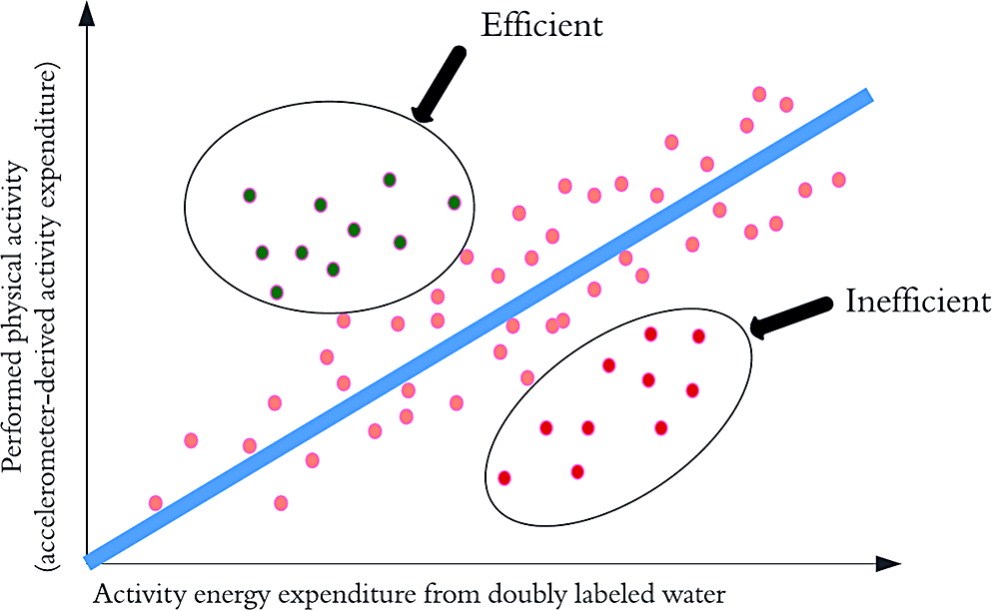
Conceptual model of our definition of movement efficiency

Physical activity is known to prevent and improve chronic health conditions in the general population [6]. However, the benefit from performing the same type and dose of physical activity is not the same across individuals and populations. One example is cardiorespiratory and muscular fitness, where exercise improves fitness, but the magnitude of response is modified typically because of genetic differences [7]. Activity monitors can estimate activity energy expenditure based on the individual’s weight and performed activity. However, actual energy expenditure is also dependent on movement efficiency, resting metabolism, distribution of fat-free mass, and fat mass [8]. The gold standard for assessing energy expenditure is doubly labeled water, but it does not include information about performed physical activity [8]. Movement efficiency is a measure of how economically movement is performed by the musculoskeletal and neurological systems and possibly explains why activity energy expenditure is paradoxically elevated in some patient populations with low levels of physical activity [9] and/or altered body habitus. This results in higher energy consumption for the same amount of performed physical activity in those with poor movement efficiency than in those with high movement efficiency, Fig. 1. Because overweight or obesity contribute to biomechanical alterations, i.e. excessive body motion, activity energy expenditure may be overestimated when accelerometry is used as a measurement tool in obese individuals [10].

Movement efficiency can be improved in cancer patients. Carter et al. demonstrated that patients with non-metastatic breast cancer had reduced energy expenditure while increasing accelerometer-assessed physical activity during a standardized walking activity, following an exercise intervention [11]. However, there are no studies of movement efficiency in survivors of childhood ALL. Treatment of childhood ALL includes several risk factors that could potentially lead to decreased movement efficiency, such as vincristine-induced neuropathy, CNS-directed therapy, systemic high-dose methotrexate, steroid-induced obesity, and disease-related physical inactivity. Hence, it is plausible that childhood ALL survivors are at increased risk for low movement efficiency. The aim of this study was to assess movement efficiency, defined by physical activity from actigraphy and energy expenditure from doubly labeled water, and risk factors associated with low movement efficiency among survivors of childhood ALL.

## Methods

This study was performed on data in the Motor Proficiency and Physical Activity in Adult Survivors of Childhood ALL (MTRPAL) study [4, 12] within the St. Jude Lifetime Cohort (SJLIFE) Study [13]. Participants gave written informed consent before participating in the study. The St. Jude Lifetime Cohort Study was approved by the Institutional Review Board at St. Jude Children´s Research Hospital, with current approval date 03/12/2023.

### Participants

The underlying eligible population were participants in SJLIFE, previously treated for childhood cancer at St. Jude Children’s Research Hospital (SJCRH) [13]. For MTRPAL, eligible participants were diagnosed with childhood ALL (< 18 years) between 1980 and 2003, without congenital neuro- musculoskeletal or cardiopulmonary impairments, and not currently undergoing cancer treatment. A comparison group, matched with survivors on age, sex, and race, was recruited from the population of friends, relatives, and family members of current patients at SJCRH (www.clinicaltrials.gov #NCT01047020) [12].

### Energy expenditure

Resting energy expenditure (REE) was evaluated with indirect calorimetry (Ultima CardiO2; MCG Diagnostics, St. Paul, MN). Participants rested in the supine position wearing a face mask attached to a low-flow pneumotach for at least 20 min prior to the 10-minute measurement of REE [14]. REE was calculated using the modified Weir Eq.  [15]. Total daily energy expenditure (TDEE) was evaluated over 1 week with the doubly labeled water (DLW) technique. This technique assesses the elimination rates of oxygen and hydrogen, resulting in a measure of carbon dioxide flux, which is proportional to total energy expenditure [16, 17], and the methods for this in the MTRPAL study have been previously described [12]. Activity energy expenditure (AEE) was calculated by subtracting both REE and the thermal effect of food (0.10 x TDEE) from TDEE. For this study, active energy expenditure was divided by 7 days to get kilocalories per day.

### Actigraphy

Total levels of physical activity during seven days were assessed with actigraphy. Participants wore a triaxial accelerometer (wGT3X- BT; ActiGraph, Pensicola FL) over the right hip during waking hours for 7 days, removing it for bathing. Accelerometers were worn during the same days when TDEE was assessed using DLW. Accelerometer-estimated AEE (aAEE) from the Freedson Combination 1998 equation were used in the analyses [18].

### Cardiopulmonary fitness

Cardiopulmonary fitness was assessed by submaximal (85% of predicted maximal heart rate) cardiopulmonary exercise testing on a treadmill, with continuous 12-lead electrocardiogram monitoring (Ultima CardiO2, MCG Diagnostics) and breath-by-breath gas analysis using a metabolic cart (Medgraphics, VT2000), to evaluate peak oxygen uptake [19].

### Muscular strength

Muscular strength was evaluated by knee extension (Nm/kg), ankle dorsiflexion, and handgrip testing (kg) [20, 21]. Knee extension strength was measured as peak torque from five repetitions at 60 degrees per second and ankle dorsiflexion at 30 and 90 degrees per second (Biodex System III, Shirley NY) [21]. Isometric sitting handgrip testing was performed with a Jamar handheld dynamometer (Sammons Preston Rolyan, Nottinghamshire, UK). The maximum value was used.

### Neuropathy

Neuropathy was evaluated with the modified Total Neuropathy Score (mTNS) [22]. This test assesses sensory and motor symptoms, by evaluating distal muscle strength, deep tendon reflexes, light touch sensation, and vibration threshold, resulting in a score ranging from 0 (no neuropathy) to 24 (severe neuropathy). As previously described, we used a score 1.5 standard deviations above the control mean (mTNS ≥ 4) to classify neuropathy [4, 12]. In a sensitivity analysis, we also categorized neuropathy as mTNS = 0, mTNS 1–4, and mTNS ≥ 5.

We analyzed genotype of the gene CEP72 (rs924607), since the homozygous “TT” genotype has been reported to be associated with persistent neuropathy in survivors of childhood ALL [23]. CEP72 genotypes (rs924607) were determined by whole-genome sequencing (WGS) or Affymetrix Single Nucleotide Polymorphism (SNP) Array 6.0 (Affymetrix SNP6.0; Affymetrix Incorporated, Santa Clara, CA, USA) [23].

### Body composition

Height in centimeters and weight in kilograms are measured with a wall-mounted stadiometer (SECA, Hanover, MD) and an electronic scale (Scale-tronix, White Plains, NY), respectively. Body mass index was calculated (kg/m2) and categorized as normal weight (20-24.9), overweight (25-29.9), or obesity (≥ 30). Fat and lean mass were measured with dual x-ray absorptiometry (QDR 4500, software version 13.3:3; Hologic, Bedford, MA) in the total-body scanning mode. Percent body fat and percent lean mass were calculated by dividing fat mass and fat-free mass by total body mass.

### Smoking and alcohol consumption

Smoking was self-reported as never smoker, or current or previous smoking. Alcohol consumption was self-reported and risky alcohol consumption defined as > 3 drinks per day or > 7 per week for women, > 4 per day or > 14 per week for men.

### Treatment variables

Childhood cancer treatment exposures are ascertained in SJLIFE from medical records as previously described [13]. The treatment exposures included in this study were cumulative doses of epipodophyllotoxins, vincristine, anthracyclines (doxorubicin equivalent dose [24]), antimetabolites, cranial radiation, and corticosteroids.

### Statistical analysis

This was an observational analysis in an existing database from the MTRPAL study. Hence, no sample size calculation was performed for the analyses described in this manuscript [25]. To calculate movement efficiency, a linear regression with AEE from actigraphy as the dependent variable, and AEE from DLW as the independent variable were used to calculate the residuals between actual and estimated energy expenditure derived from accelerometry. The regression was performed in the full study population including both survivors and controls and movement efficiency was subsequently categorized as low (residuals <-1.3 SD), normal (-1.3 SD ≤ residuals ≤ 1.3 SD), or efficient (residuals > 1.3 SD) movement efficiency. This means that 9.68% of the full study population (survivors and controls) was categorized as inefficient and 9.68% was classified as having high movement efficiency. This threshold was chosen balancing the need for enough participants in these groups, while also using a definition that corresponds to outliers. Means (SD) were calculated for all the predictor variables in survivors, categorized as low, normal, and efficient survivors. The covariates available for inclusion in the analyses were selected according to a directed acyclic graph for each analysis, created using www.dagitty.net, Supplementary Fig. 1. We considered logistic regression adjusted only for BMI category to assess the risk of low movement efficiency in survivors compared to controls because controls were matched to survivors by sex, race, and gender [12]. Two separate logistic regressions, with variables chosen using elastic net [26], were used to determine if treatment (model 1) or lifestyle/functional measures (model 2) were associated with being categorized as low compared to normal and efficient. Treatment exposures examined for selection in model 1 included: craniospinal radiation treatment, methotrexate, vincristine, alkylating agents, anthracyclines (doxorubicin equivalent dose [24]), epipodophyllotoxins, and corticosteroids. Variables examined for selection in model 2 included: gender, race, BMI-defined overweight and obesity, neuropathy (modified total neuropathy score) [4, 22], knee extension strength (nm/kg), aerobic capacity (VO_2_ max), percent lean mass, and smoking status. This model was also performed for the control population to confirm associations between BMI status and movement efficiency. We further aimed to assess whether the association between vincristine exposure and movement efficiency was mediated by *CEP72* genotype [23].

## Results

The study included 256 controls and 302 survivors, 48% female with mean age at ALL diagnosis of 7 years and mean age at assessment of 29 years. All 302 survivors had been exposed to some dose of both vincristine and corticosteroids and 131 (43%) had been exposed to cranial radiation therapy (Table 1). For movement efficiency, 24 survivors were categorized as efficient, 245 as normal, and 33 as inefficient, with distributions illustrated in Supplementary Fig. 2. There was no difference in the prevalence of low movement efficiency between survivors (*n* = 33, 10.9%) compared to controls (*n* = 23, 9.0%) or risk of being classified as having normal (odds ratio [OR]: 1.19, 95% confidence interval [CI] 0.67–2.10, *p* = 0.55) or high movement efficiency (OR: 1.91, 95% CI 0.87–4.20, *p* = 0.11).

**Table 1:** Baseline demographics by movement efficiency category

Variable	Inefficient (*n* = 33)	Normal (*n* = 245)	Efficient (*n* = 24)
Age (years) mean (sd)	29.4 (6.7)	29.0 (5.9)	30.3 (5.8)
Age at diagnosis (years), mean (sd)	7.3 (4.5)	7.0 (4.7)	6.2 (4.5)
Gender, n (%)			
Male	14 (42.4)	128 (52.2)	14 (58.3)
Race, n (%)			
White	28 (84.8)	214 (87.3)	22 (91.7)
Other	5 (15.2)	31 (12.7)	2 (8.3)
Unemployed, n (%)	14 (42.4)	61 (24.9)	4 (16.7)
Smoker, n (%)			
Past	1 (3.03)	27 (11.0)	4 (16.7)
Current	11 (33.3)	58 (23.7)	4 (16.7)
Never	21 (63.6)	160 (65.3)	16 (66.6)
Body mass index (kg/m2), mean (sd)	27.5 (6.4)	28.4 (8.9)	32.1 (8.1)
Normal weight, n (%)	12 (36.4)	85 (34.7)	7 (29.2)
Overweight, n (%)	12 (36.4)	73 (29.8)	3 (12.5)
Obesity, n (%)	9 (27.2)	87 (35.5)	14 (58.3)
Body fat %, mean (sd)	31.2 (9.4)	31.4 (8.9)	32.4 (9.7)
Lean mass %, mean (sd)	68.7 (9.4)	68.5 (8.9)	67.5 (9.7)
Knee extension strength (nm/kg), mean (sd)	168.4 (68.4)	173.9 (54.9)	169.8 (57.2)
Ankle strength (nm/kg), mean (sd)	22.9 (9.9)	25.5 (9.7)	24.9 (9.7)
Peak vo2 (ml/kg/min), mean (sd)	21.8 (4.8)	24.2 (6.2)	24.5 (6.6)
Neuropathy score (mtns), mean (sd)	3.4 (2.4)	2.8 (2.2)	2.4 (1.9)
Neuropathy, n (%)	17 (51.5)	91 (37.1)	8 (33.3)
Epipodophyllotoxins, n (%)	27 (81.8)	213 (86.9)	20 (83.3)
Etoposide, n (%)	19 (57.6)	149 (60.8)	10 (41.7)
Median (range), mg/m2	9,224 (798 − 15,821)	9,511 (400 − 23,630)	9,846 (614 − 15,438)
Teniposide, n (%)	17 (51.5)	153 (62.4)	17 (70.8)
Median (range), mg/m2	3,408 (452-7,284)	3,214 (150 − 10,339)	3,447 (592-7,234)
Anthracyclines, n (%)	26 (78.8)	180 (73.5)	14 (58.3)
Median (range), mg/m2	49.8 (12.2-270.3)	38.2 (19.8-348.3)	51.2 (25.0-236.4)
Corticosteroids, n (%)	33 (100.0)	245 (100.0)	24 (100.0)
Prednisone			
≤5,000 mg/m2	15 (45.5)	128 (52.2)	14 (58.3)
5,001–15,000 mg/m2	16 (48.5)	102 (41.6)	9 (37.5)
>15,000 mg/m2	2 (6.1)	15 (6.1)	1 (4.2)
Dexamethasone			
Median (range), mg/m2	1,568 (280-2,324)	1,568 (72 − 12,880)	1,812 (1,344-2,280)
Antimetabolites, n (%)			
IV methotrexate	28 (84.8)	213 (86.9)	18 (75.0)
Median (range), mg/m2	15,065 (449 − 36,749)	14,370 (899 − 40,571)	11,579 (3,488 − 24,486)
High dose methotrexate, n (%)	27 (81.8)	212 (86.5)	18 (75.0)
IT methotrexate			
Median (range), mg/m2	160.7 (9.2-316.4)	192.9 (57.1-721.5)	162.9 (74.5–419.0)
Vincristine			
Median (range), mg/m2	46.5 (4.4–63.6)	45.7 (3.3-102.7)	9.2 (4.0-65.6)
>39 mg/m2 n, (%)	18 (54.5)	140 (57.1)	10 (41.7)
Cranial radiation, n (%)	13 (39.4)	107 (43.7)	11(45.8)

In the model of associations between treatment exposures in survivors and subsequent movement efficiency, no treatments were selected by the elastic net procedure, Table 2. For the functional assessments model, neuropathy was associated with higher risk of being inefficient compared to efficient (odds ratio 4.30, 95% confidence interval (CI) 1.03–17.96), Table 2. Obesity (≥ 30 kg/m^2^) had a protective association (odds ratio 0.18, 95% CI 0.04–0.87), while the estimate for overweight indicated a non-significant higher risk of poor movement efficiency for survivors with overweight (odds ratio 1.77, 95% CI 0.35–9.06), Table 2. Supplementary Table 1 shows that obesity was associated with lower risk of poor movement efficiency in controls as well (odds ratio 0.078, 95% CI 0.012–0.499). Supplementary Table 2 shows that ALL survivors categorized as efficient had the highest prevalence of no neuropathy (25%), while survivors with normal movement efficiency had the highest prevalence of an mTNS score 1–4 (61%), and survivors with poor movement efficiency had the highest prevalence of an mTNS score ≥ 5 (27%). The model for associations between function/lifestyle measures and risk of being inefficient compared to normal resulted in a model with no variables included, Table 2. There were no significant differences in the distribution (CC, CT, TT) of *CEP72* genotypes [23] among survivors who were inefficient (48%, 42%, 10%), normal (40%, 43%, 16%), or efficient (27%, 64%, 9%).

**Table 2:** Risk factors for low movement efficiency compared to having normal or high movement efficiency

Model 1: Treatment variables^1^		
*Risk of being inefficient compared to normal*		
No variables selected by elastic net		
*Risk of being inefficient compared to efficient*		
No variables selected by elastic net		
Model 2: Variables from functional assessment^2^		
*Risk of being inefficient compared to normal*		
No variables selected by elastic net		
*Risk of being inefficient compared to efficient*		
**Exposure**	**Odds ratio**	**95% confidence interval**
Neuropathy	4.30	1.03–17.96
Overweight	1.77	0.35–9.06
Obesity	0.18	0.04–0.87

^1^Variables available for selection in the elastic net were exposure to craniospinal radiation treatment, methotrexate, vincristine, alkylating agents, anthracyclines, epipodophyllotoxins, and corticosteroids. ^2^Variables available for selection in the elastic net were gender, race, smoking status, BMI category (overweight & obesity), lean mass, knee extension strength, aerobic capacity, and neuropathy.

## Discussion

We report results from the first study of movement efficiency in survivors of childhood ALL. By defining movement efficiency with correlations between energy expenditure assessed with actigraphy and doubly labeled water, this study identified an association of neuropathy with a higher risk for inefficient movement in these survivors. Interestingly, obesity appeared to be protective for poor movement efficiency. These results further highlight impairments associated with treatment-induced neuropathy in survivors of childhood ALL.

Movement efficiency is a measure of how effectively activity can be performed by the combined neuromusculoskeletal system, and neuropathy is an impairment of the neurological part of that system. The association between neuropathy and movement efficiency in our study was anticipated. Since neuropathy in ALL survivors is mostly caused by vincristine exposure, we had anticipated that this would translate to an association between vincristine and movement efficiency. There are several possible explanations for the lack of associations found in our study. One is the relatively limited sample size, but another is the fact that all survivors were exposed to some dose of vincristine. It is possible that poor movement efficiency only occurs in those who develop neuropathy due to a combination of vincristine exposure and genetic sensitivity to vincristine [23], rather than in all individuals exposed to higher doses of vincristine. However, our sensitivity analyses could not confirm mediation by genetic status, possibly due to insufficient statistical power. Before the analyses were performed, we also hypothesized that movement efficiency would be associated with poor knee extension strength, caused by higher doses of corticosteroids leading to proximal myopathy [27]. However, our analyses showed no associations between corticosteroid exposures nor knee extension strength and movement efficiency, which might indicate that impairments in movement efficiency in ALL survivors are caused more by neuropathy than myopathy.

Given the fact that ALL survivors have been exposed to several treatments that could lead to reduced movement efficiency, in terms of both neurological deficits from CNS-directed therapy, vincristine-induced neuropathy, and corticosteroid-induced myopathy, it was somewhat surprising that there were no significant differences in the risk for low movement efficiency between survivors and controls. One possible explanation is that obesity, which is prevalent among ALL survivors [28, 29], was seemingly protective for risk of poor movement efficiency. This was also the case for the control population. This may be related to actigraphy overestimating the level of physical activity due to excessive body motion. This measurement error has been previously described in individuals with obesity [10]. It has also been reported that doubly labeled water may underestimate energy expenditure in individuals with obesity [30], which could further explain the discordance between AEE from actigraphy and doubly labeled water in our participants with obesity. Our interpretation of underestimated AEE from actigraphy in participants with obesity is further supported by the analyses indicating a (non-significant) higher odds ratio for poor movement efficiency in survivors with overweight i.e., no dose-response association.

### Definitions of movement efficiency

Movement efficiency is generally defined as the ratio of the mechanical work performed and the metabolic cost of performing that work [31]. This can be done in several ways, usually in a controlled laboratory setting where both energy consumption and the performed physical activity are standardized [32]. This results in e.g. an measure of the energetic cost of walking, a measure of movement efficiency. Using this definition, it has been shown that exercise interventions can reduce the energetic cost of walking in an elderly population, corresponding to improved movement efficiency [33] While assessments in a laboratory increases the validity of the assessment, it reduces the applicability of the results to what participants are performing in everyday life. In our definition, we have used the gold standard for assessing free-living energy consumption, doubly labeled water, and combined it with the most appropriate assessment of free-living physical activity, actigraphy. While we aren´t aware of other studies that have defined movement efficiency using our combination of actigraphy and doubly labeled water, the fact that movement efficiency modifies the relationship between performed activity and AEE has led to the development of methods for adjusting free-living AEE for movement efficiency, using individual assessments of energy expenditure during standardized tasks [34]. The activity-related time equivalent (ARTEEE) corrects AEE for the economy of performing standardized physical activities performed in a laboratory setting [34]. While the ARTEEE handles bias from movement efficiency when assessing AEE, it does not directly result in a measure of movement efficiency and is resource consuming. There are also previous studies of unexpectedly high energy expenditure in patients with chronic obstructive pulmonary disease [9], and this has been explained with a higher energy consumption for producing the same movements [8], corresponding to our definition of poor movement efficiency.

For movement efficiency in cancer populations, Carter et al. assessed the effects of an exercise intervention on the energetic cost of walking in breast cancer survivors, with assessments during a standardized walking task in a laboratory setting [11]. Performed physical activity was assessed with actigraphy movement counts, during a standardized treadmill walking task. Energy consumption was assessed with indirect calorimetry. Survivors in the intervention group demonstrated decreased energetic cost of walking, assessed by lower results in indirect calorimetry, while increasing their physical activity during walking, explained by more vertical accelerations during the same standardized walking task. They concluded that hip-worn accelerometers aren´t well-suited to detect changes in walking efficiency. However, using our definition of movement efficiency, both increased activity and reduced energy expenditure would have resulted in higher movement efficiency. This further highlights that different definitions of movement efficiency have built-in strengths and limitations and may yield different results.

### Strengths and limitations

This study has several strengths. One is the assessment of movement efficiency in free-living physical activity as opposed to during standardized tasks. This increases the applicability of the results to all aspects of daily living. However, the lack of a standardized task reduces the ability to control for measurement errors in actigraphy, as seen in our results for obesity. Another strength is the use of the gold standard doubly labeled water for assessing energy expenditure and the simultaneous assessment with actigraphy, enabling correlations between the two measures. The fact that the study was performed within SJLIFE, with comprehensively ascertained treatment exposures and clinical functional assessments, further increases the validity of the results. A main limitation of the study is the relatively limited sample size, reducing statistical power due to a low number of participants categorized as efficient/inefficient. It is possible that this has led to type II errors where actual associations between treatment exposures, functional parameters, and movement efficiency could not be detected.

In conclusion, neuropathy was associated with higher risk of poor movement efficiency in survivors of childhood ALL. These results further highlight impairments associated with treatment-induced neuropathy in survivors of childhood ALL and indicate that survivors who suffer from persistent neuropathy consume more energy while performing the same type of physical activity as survivors without neuropathy.

## Electronic supplementary material

Below is the link to the electronic supplementary material.

Supplementary Material 1

Supplementary Material 2

Supplementary Material 3

## Data Availability

The data that support the findings of this study are openly available at: https://www.stjude.cloud/research-domains/cancer-survivorship. Data specific for this paper will be uploaded to https://zenodo.org concomitant with publication of the manuscript.
