# Supplementary figures and images for: Movement efficiency in survivors of childhood acute lymphoblastic leukemia: a report from the St. Jude lifetime cohort study
Source: J Cancer Surviv. 2024 Feb 3;19(4):1264–71. doi: 10.1007/s11764-024-01550-1 (PMC11297192; doi:10.1007/s11764-024-01550-1)

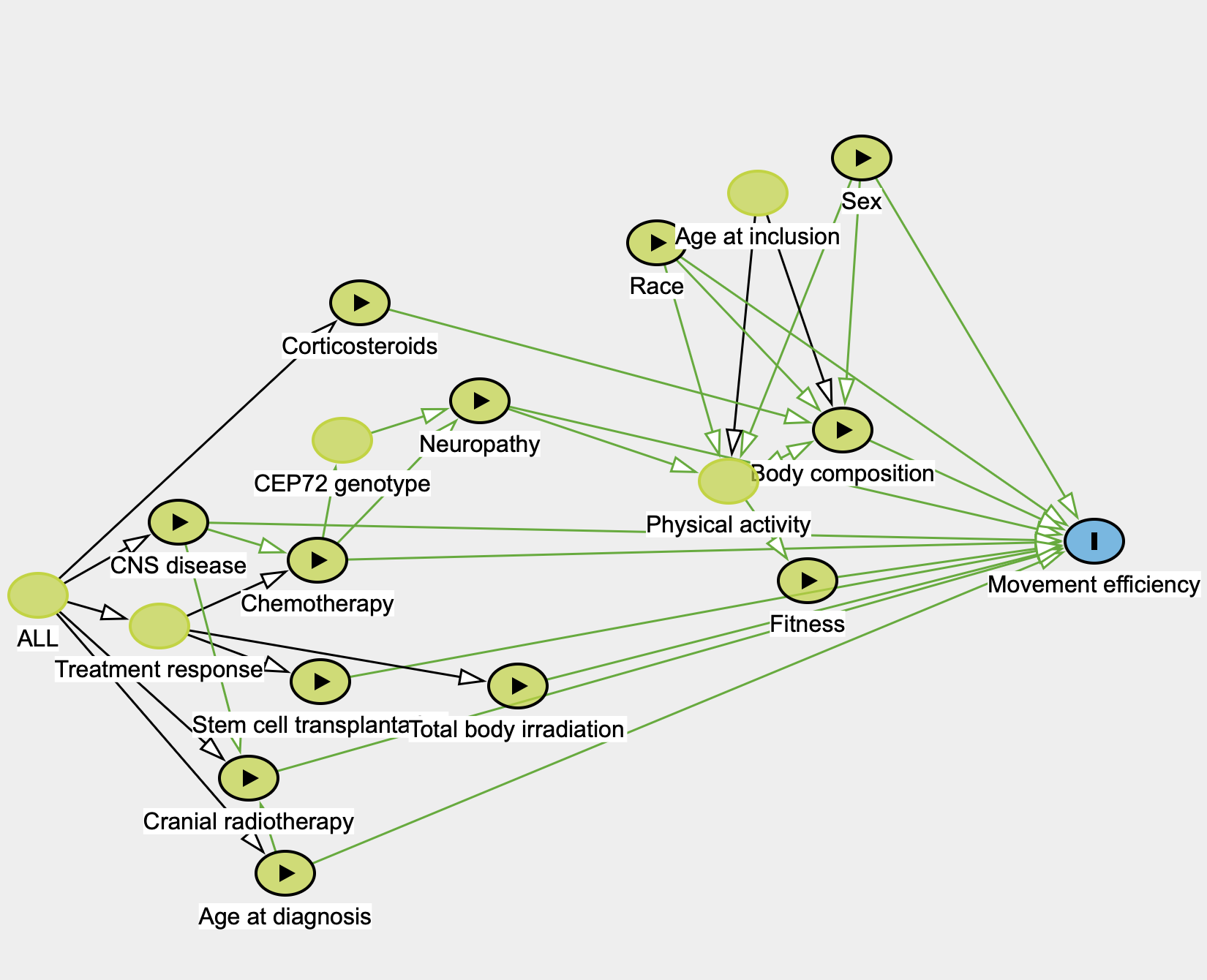

Supplement: Supplementary file 1 — Supplementary Material 1 [file 11764_2024_1550_MOESM1_ESM.png]

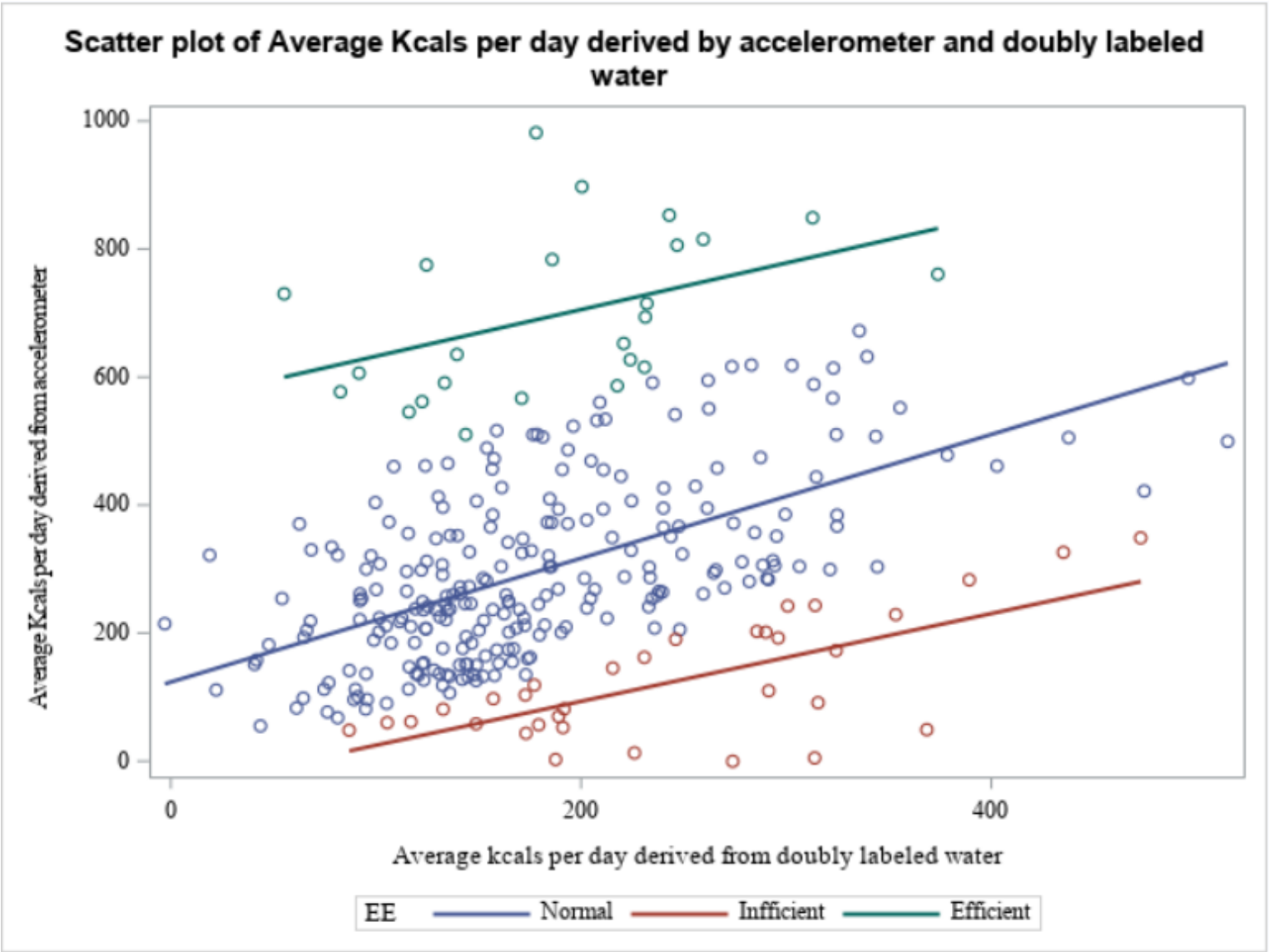

Supplement: Supplementary file 2 — Supplementary Material 2 [file 11764_2024_1550_MOESM2_ESM.pdf]
