# Supplementary material for: Movement efficiency in survivors of childhood acute lymphoblastic leukemia: a report from the St. Jude lifetime cohort study
Source: J Cancer Surviv. 2024 Feb 3;19(4):1264–71. doi: 10.1007/s11764-024-01550-1 (PMC11297192; doi:10.1007/s11764-024-01550-1)
Supplement: Supplementary file 3 — Supplementary Material 3 [file 11764_2024_1550_MOESM3_ESM.docx]

Supplementary table 1. Risk factors for low movement efficiency compared to having normal or high movement efficiency in controls.

| *Risk of being inefficient compared to normal* | | |
| --- | --- | --- |
| No variables selected by elastic net | | |
| *Risk of being inefficient compared to efficient* | | |
| Exposure | **Odds ratio** | **95% confidence interval** |
| Female | 5.81 | 1.33-25.35 |
| Overweight | 0.075 | 0.012-0.448 |
| Obesity | 0.078 | 0.012-0.499 |
| Non-white | 0.14 | 0.012-1.71 |

Variables available for selection in the elastic net were gender, race, smoking status, BMI category (overweight & obesity), lean mass, knee extension strength, aerobic capacity, and neuropathy.

Supplementary table 2. Prevalence of no, moderate, or severe neuropathy by movement efficiency category.

| Variable | Inefficient (n = 33) | Normal (n = 245) | Efficient (n = 24) |
| --- | --- | --- | --- |
| Neuropathy, n (%) |  |  |  |
| mTNS=0 | 6 (18.2) | 49 (20.1) | 6 (25.0) |
| mTNS 1-4 | 18 (54.6) | 149 (61.1) | 13 (54.2) |
| mTNS ≥5 | 9 (27.3) | 46 (18.9) | 5 (20.8) |

mTNS=modified total neuropathy score.
